# RNF166 promotes colorectal cancer progression by recognizing and destabilizing poly-ADP-ribosylated angiomotins

**DOI:** 10.1038/s41419-024-06595-9

**Published:** 2024-03-13

**Authors:** Yun Li, Xiangqian Zhang, Na Liu, Ruijie Liu, Wuming Zhang, Lin Chen, Yongheng Chen

**Affiliations:** 1 Department of Oncology, NHC Key Laboratory of Cancer Proteomics & State Local Joint Engineering Laboratory for Anticancer Drugs, National Clinical Research Center for Geriatric Disorders, Xiangya Hospital, Central South University, Changsha, 410008 Hunan China; 2 Department of Endocrinology, Xiangya Hospital, Central South University, Changsha, 410008 Hunan China; 3 Department of Pathology, Xiangya Hospital, Central South University, Changsha, 410008 Hunan China; 4 Department of General Surgery, Xiangya Hospital, Central South University, Changsha, 410008 Hunan China

**Keywords:** Oncogenes, Colorectal cancer, Cell signalling

## Abstract

Activation of the Hippo pathway by angiomotins to limit colorectal cancer progression is prevalent, whereas the regulation of angiomotins remains elusive. In this study, we uncover the involvement of an upregulated E3 ubiquitin ligase called RNF166, which destabilizes angiomotins, activates YAP, and is associated with a poor prognosis in colorectal cancer patients. Mechanistically, RNF166 specifically recognizes PARsylated angiomotin, a modification mediated by tankyrase at specific amino acid residues (D506, E513, E516, and E528). The tankyrase inhibitor XAV939, effectively prevents RNF166-dependent destabilization of angiomotins and subsequent activation of YAP. Additionally, YAP-5SA, a constitutively active form of YAP, rescues colorectal cancer progression following knockdown of RNF166. Importantly, the C-terminus of RNF66, particularly the Di19-ZF domain, is the crucial region responsible for recognizing ADP-ribosylated angiomotins. Together, this work not only sheds light on the regulation of the Hippo pathway in colorectal cancer but also uncovers a novel poly(ADP-ribose)-binding domain, which may serve as a potential therapeutic target for intervention.

## Introduction

Colorectal cancer (CRC) is a significant global health concern, ranking as the third most diagnosed cancer and the second leading cause of cancer-related deaths worldwide [1, 2]. Colon adenocarcinoma (COAD) is the dominant type of colorectal cancer. The high mortality in CRC is primarily attributed to advanced diagnoses and resistance to conventional treatments, which are linked to dysregulated signaling pathways [3]. One key regulatory factor involved in intestinal epithelium regeneration and CRC malignancy is the transcriptional coactivator called yes-associated protein (YAP), which acts as the downstream effector of the Hippo pathway. Activation of the Hippo pathway, also known as YAP inactivation, involves YAP retention in the cytoplasm, phosphorylation at S127 and degradation, preventing YAP from transcribing growth factors, such as CYR61 and CTGF [4, 5]. The Hippo pathway is primarily regulated by the kinase cascade pathway, tight junctions, and cell polarity proteins, including angiomotin (AMOT) family proteins (Motins) and Merlin/Neurofibromin (NF2). The Motins consist of three members: AMOT, AMOTL1, and AMOTL2. Two isoforms of AMOT, AMOTp80 and AMOTp130, exist due to alternative splicing. AMOTp80 lacks the N-terminal region present in AMOTp130. Through the PPXY motif located in their N-terminal regions, Motins specifically bind to YAP/TAZ at cell junctions, sequestering YAP and preventing its nuclear translocation [6]. Furthermore, Motins promote YAP phosphorylation by activating upstream LATS1/2 autophosphorylation, thereby indirectly modulating the Hippo pathway [7, 8]. Thus, the regulation of Motins can indirectly affect the Hippo pathway. For instance, tankyrase (TNKS), also known as poly(ADP-ribose) polymerase 5 (PARP5), affects YAP activation by recruiting the E3 ubiquitin ligase RNF146, which destabilizes Motins [9]. However, how Motins are modified by TNKS and whether other E3 ubiquitin ligases are recruited in TNKS-mediated Motins degradation remains unknown.

In addition to RNF146, another E3 ubiquitin ligase, RNF166, was simultaneously identified through prey baiting by TNKS [9]. RNF166 belongs to the RING-type E3 ligase family [10] and has been associated with various physiological processes, including antibacterial autophagy [11], interferon production induced by RNA viruses [12], and proapoptotic effects in the central nervous system [13]. Its biological functions are closely tied to its protein structure. RNF166 comprises a RING domain and a C2HC Zn finger (ZF) domain at the N-terminus, a conserved drought-induced 19 zinc-binding (Di19-ZF) domain, and a ubiquitin-interacting motif (UIM) motif at the C-terminus [14]. The Di19 domain was initially discovered as responsive to drought in Arabidopsis Abscisi [15], but its function remains unknown. Given the limited knowledge on RNF166, its role in tumorigenesis, particularly in CRC, remains unexplored.

Ubiquitination is regulated by factors such as substrate modifications, small molecules, binding partners, and substrate competition [16]. Ubiquitination directed by ribosylation modification is implicated in telomere dynamics, transcription, metabolism, and tumor signaling [17]. Ribosylation occurs when ADP-ribosyl transferases (PARPs in mammals) add ADP to protein receptors or DNA/RNA ends, resulting in mono-ribosylation or poly-ribosylation (PARsylation). The receptor residues are diverse, including glutamate (E), aspartate (D), serine (S), arginine (R), lysine (K), or cysteine (C) amino acids [18]. Tankyrase, responsible for PARsylation, exerts multiple oncogenic effects by promoting telomere elongation [19], maintaining cell survival following telomeric DNA damage [20], and participating in tumor-related signaling pathways such as the Hippo signaling pathway [9], LKB1-AMPK pathway [21], and Notch signaling [22]. Selective PARP inhibitors (e.g., olaparib) have been approved for the treatment of triple-negative breast cancer [23], and TNKS inhibitors also hold therapeutic potential [24].

In this study, we uncover the role of RNF166 in promoting CRC tumor growth through the recognition and degradation of PARsylated Motins, leading to YAP activation. This process is dependent on TNKS. Importantly, the C-terminus of RNF166, particularly the Di19-ZF domain, is a novel poly(ADP-ribose)-binding domain (PBD).

## Results

### RNF166 is upregulated in CRC and promotes tumor progression

**Fig. 1:**
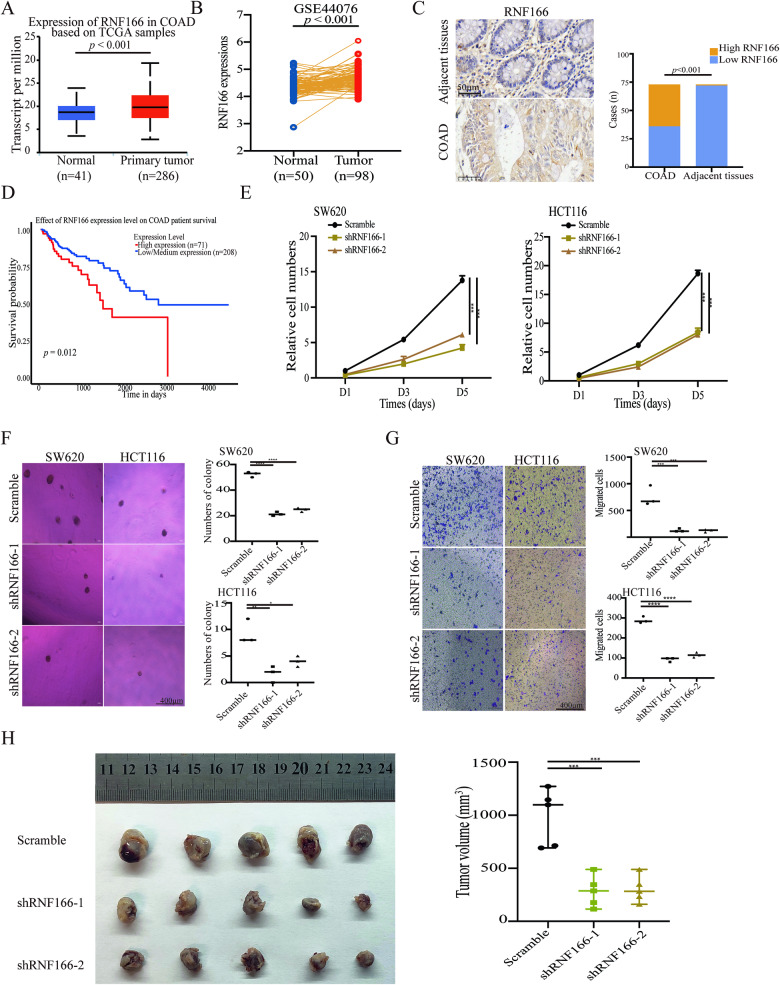
Upregulated RNF166 promotes CRC progression. RNF166 expression in normal colon tissue and colon cancer tissue from the UALCAN website (**A**) and paired samples of COAD from the GSE44076 dataset (**B**). **C** Immunohistochemistry showing RNF166 expression in 73 paired human CRC samples. The representative image is on the left, and statistical analysis of positive cases is on the right (*χ*^2^ = 96.019, *p* < 0.001). **D** Kaplan–Meier survival curves showing the association between tumor RNF166 levels and the probability of survival in CRC patient cohorts from UALCAN websites (*p* = 0.012). Proliferation (**E**), anchorage-independent growth (**F**), and migration ability (**G**) of HCT116 and SW620 cells with or without RNF166 knockdown. **H** HCT116 cells stably transfected with RNF166 shRNA and control were subcutaneously inoculated into immunodeficient mice. The xenograft tumors were resected and measured at the final timepoint, with 5 mice per cohort. For (**E**−**H**), *p < 0.05, **p < 0.01, ***p < 0.001, and ****p < 0.0001.

To determine the role of RNF166 in CRC, we examined its expression in colon adenocarcinoma and paracancerous tissues. Significantly higher expression of RNF166 in COAD tissues than in normal tissues was observed in UALCAN, which provided easy access to TCGA data (Fig. 1A) [25]. In addition, GSE44076 [26] also revealed upregulated RNF166 in COAD (Fig. 1B). Immunohistochemistry (IHC) staining further confirms positive RNF166 expression in COAD tumors and minimal expression in normal intestinal glands epithelium in 73 pairs of COAD tissues (Fig. 1C). Additionally, survival analysis reveals that patients with high RNF166 expression have poorer outcomes in COAD (Fig. 1D).

Considering the significantly upregulated RNF166, RNF166 may contribute to COAD progression. To test our hypothesis, we depleted RNF166 using shRNA in the human CRC-derived cell lines SW620 and HCT116 (Fig. S1A, S1B). Reducing RNF166 expression leads to a significant decrease in cell proliferation (Fig. 1E), anchorage-independent growth (Fig. 1F), and cell migration (Fig. 1G) in both cell lines. Furthermore, the subcutaneous transplantation tumor model in immunodeficient mice showed that RNF166 knockdown (KD) inhibits tumor growth in vivo (Fig. 1H). These results suggest that RNF166 upregulation contributes to COAD progression.

### RNF166 strongly interacts with Motins

**Fig. 2:**
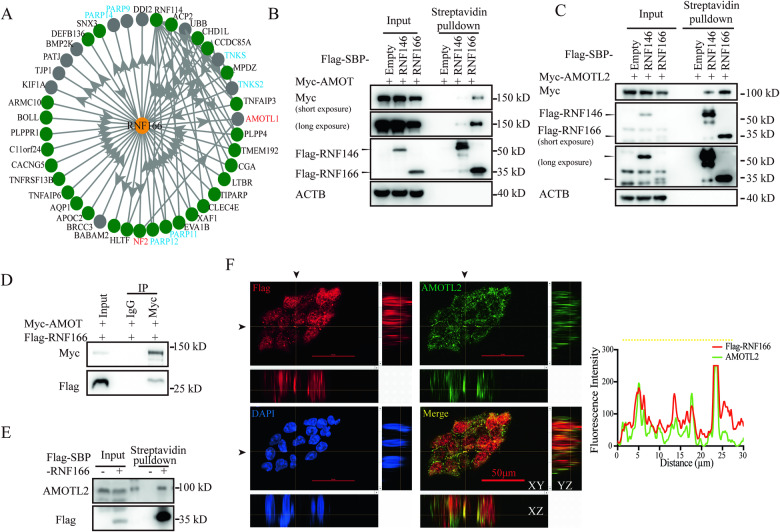
Identification of Motins as RNF166 interacted protein. **A** Protein–protein interaction network of RNF166 from the Bioplex interactome database. Interplay between RNF166 and Motins. 293 T cells (**B**−**D**) or HCT116 cells (**E**) transfected with the indicated plasmids. Cell extracts were incubated with streptavidin agarose (**B**, **C**, **E**) or immunoprecipitated with normal mouse IgG/anti-Myc antibody (**D**). **F** Orthogonal projections illustrating the colocalization of RNF166 (red) and AMOTL2 (green). Indicated (yellow) intensity trace is plotted at right. HCT116 cells were stably overexpressed Flag-RNF166. Samples were coimmunized with mouse anti-Flag and rabbit anti-AMOTL2 antibodies and incubated with goat anti-mouse and goat anti-rabbit secondary antibodies. DNA was stained with DAPI (blue). Scale bar, 50 μm. Abbreviations: DAPI, 2-(4-Amidinophenyl)-6-indolecarbamidine dihydrochloride.

Next, we tested how RNF166 promoted COAD progression. Given that RNF166 functions as an E3 ubiquitin ligase, we aim to identify its interacting proteins. Through analysis of the Bioplex interactome database [27], 48 proteins are identified as potential interactors of RNF166 (Fig. 2A, Supplementary Table 1). The KEGG enrichment analysis within KOBAS [28] revealed that these interacting proteins are primarily associated with tight junctions, including NF2, AMOT, AMOTL1, PATJ, MPDZ and TJP1. These proteins are also involved in the Hippo signaling pathway, which contains PATJ, NF2 and AMOT (Fig. S2). The Hippo signaling pathway is critical for tumorigenesis and regulates tumor cell proliferation and invasion, which agrees with the phenotype observed following RNF166 knockdown.

We focused on Motins and NF2 because there is a direct interaction between them, and both proteins are scaffolds in the Hippo signaling pathway [29, 30]. We then tested their interplay with RNF166. Streptavidin binding peptide (SBP) and FLAG tandem tagged RNF166 was coexpressed with its potential interacting proteins in 293 T cells. A strong interaction between RNF166 and Motins (AMOT and AMOTL2) was observed (Fig. 2B, C), while the interaction between RNF166 and NF2 was relatively weak (Fig. S3A). Interestingly, the interaction between RNF166 and AMOT was even stronger than that of RNF146, a known ubiquitin ligase involved in Motins degradation [9]. Immunoprecipitation experiments demonstrated that AMOT could pull down RNF166 (Fig. 2D). Meanwhile, their interaction was also observed in HCT116 cells (Fig. 2E). Immunofluorescence revealed co-localization of RNF166 with AMOT (Fig. S3B) and AMOTL2 (Fig. 2F), especially AMOTL2, which was the most abundant Motins in colon cancer cells (Fig. S4). These results indicate that RNF166 strongly interacts with Motins.

### RNF166 mediates Motins polyubiquitination and degradation

**Fig. 3:**
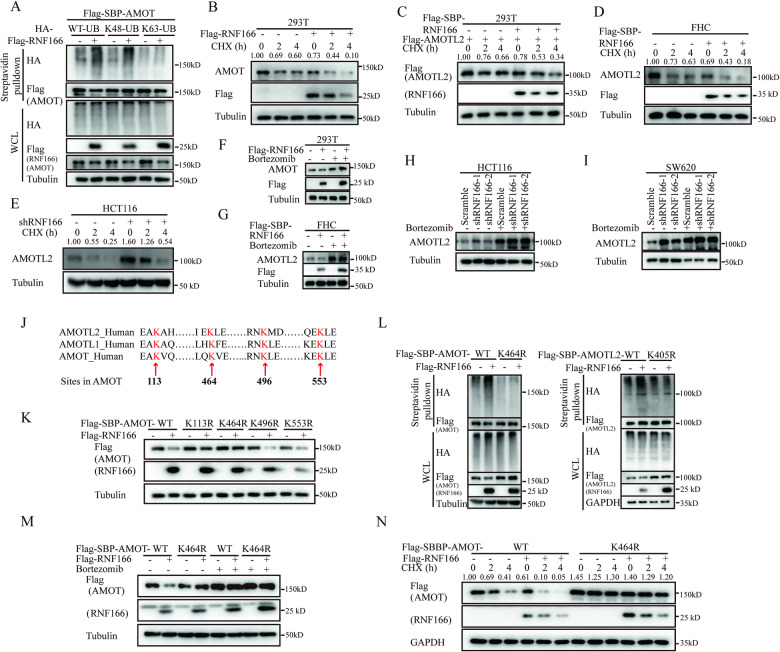
RNF166 mediates the polyubiquitination and proteasomal degradation of Motins. **A** RNF166 mediates K48-linked polyubiquitination of AMOT. 293 T cells were cotransfected with Flag and SBP-tagged AMOT, a vector encoding HA-Ub-WT or its mutants (HA-Ub-K48 or HA-Ub-K63), and Flag-RNF166 or empty vector for 48 h. Cell lysates were incubated with streptavidin agarose for 3 h and immunoblotted with the indicated antibodies. **B**−**E** RNF166 regulates the stability of Motins. Motins protein levels were detected by immunoblots after treatment with CHX in 293 T cells transfected with Flag-RNF166 (**B**, **C**), FHC cells stably overexpressing Flag and SBP-tagged RNF166 (**D**), and HCT116 cells stably transfected with RNF166 shRNA (**E**). **F**−**I** RNF166 mediates proteasomal degradation of Motins. The effect of bortezomib treatment (0.1 μM, 12 h) on endogenous Motins were detected in 293 T cells transfected with Flag-RNF166 (**F**), FHC cells stably overexpressing Flag and SBP-tagged RNF166 (**G**), and CRC cells stably transfected with shRNF166 (**H**, **I**). **J** Possible ubiquitinated residues in Motins (red arrows) by combining ubiquitination sites from the PhosphoSitePlus database with conserved lysine residues. The conserved amino acids were compared with ClustalW. **K** Effect of RNF166 on the expression level of WT and mutant AMOT. 293 T cells were transfected with the indicated plasmids at a 1:1 ratio for 48 h and cell samples were prepared for further immunoblotting analysis. **L** RNF166 did not increase the ubiquitination of AMOT K464R or AMOTL2 K405R compared to wild-type Motins. 293 T cells were transfected with the corresponding plasmids and then treated as in (**A**). AMOT K464R was resistant to RNF166-induced degradation with or without CHX (**M**) and bortezomib (**N**) treatment. Abbreviations: CHX cycloheximide, K lysine, R arginine, SBP streptavidin-binding peptide, Ub ubiquitin, WT wild-type.

As an E3 ligase, RNF166 can recognize specific substrates and catalyze their ubiquitination. RNF166 overexpression significantly led to a significant increase in K48-linked ubiquitination of AMOT rather than K63-linked ubiquitination (Fig. 3A), indicating that AMOT was a substrate for RNF166. K48-linked polyubiquitination is commonly associated with protein degradation through the proteasome [16]. Therefore, we examined the effect of RNF166 on Motins stabilization. Exogenous expression of AMOT and AMOTL2 decreased following RNF166 overexpression (Fig. 2B, C). Although NF2 interacted with RNF166, NF2 expression increased after RNF166 overexpression (Fig. S3). This observation aligns with a previous study suggesting that Motins can act as scaffolds mediating NF2 degradation [29]. Therefore, elevated NF2 may be a result of Motins degradation. Furthermore, we also assessed the endogenous Motins affected by RNF166. Overexpression of RNF166 significantly reduced the half-life of Motins in the presence of the mRNA translation inhibitor cycloheximide (CHX) in 293 T and FHC cells (Fig. 3B−D). Conversely, deprivation of RNF166 in HCT116 cells increased the cellular level of Motins (Fig. 3E). To demonstrate RNF166-mediated Motins degradation through the proteasome, the proteasome inhibitor bortezomib [31] was utilized. Bortezomib treatment effectively restored Motins after RNF166 overexpression in 293 T and FHC cells (Fig. 3F, G), while RNF166 knockdown did not further increase Motins expression following bortezomib treatment (Fig. 3H, I). Taken together, these results suggest that RNF166 mediates K48-linked polyubiquitination and degradation of Motins through the proteasome.

We then attempted to map the ubiquitination sites on Motins. Our preliminary results suggested that RNF166 recognized and degraded Motins; therefore, these ubiquitination sites should be commonly conserved lysines in Motins. We next searched the PhosphoSitePlus database [32] to determine the conserved ubiquitination sites of Motins (Fig. S5A). Four potential lysine sites in AMOT, including K113, K464, K496 and K553, were identified (Figs. 3J and S5B). Subsequent analysis showed that only the mutation of AMOT at the K464 site (AMOT K464R) was still stable even after RNF166 overexpression (Fig. 3K). Ubiquitination assays further confirmed that RNF166 no longer promoted the ubiquitination of AMOT K464R compared to wild-type AMOT (Fig. 3L). Moreover, the expression and stability of AMOT K464R were significantly higher than that of wild-type AMOT and remained unaffected by RNF166 overexpression and bortezomib treatment by immunoblotting (Fig. 3M, N). Similar results were observed by immunofluorescence (Fig. S5C). Similarly, the AMOTL2 K405R mutant, equivalent to AMOT K464R, was also not ubiquitinated or degraded by RNF166 (Fig. 3L, Fig. S5D and S5E). In summary, these findings indicate that RNF166 mediates K48-linked polyubiquitination and degradation of Motins, specifically targeting the K464 site in AMOT and the K405 site in AMOTL2.

### TNKS is required for the regulation of Motins by RNF166

**Fig. 4:**
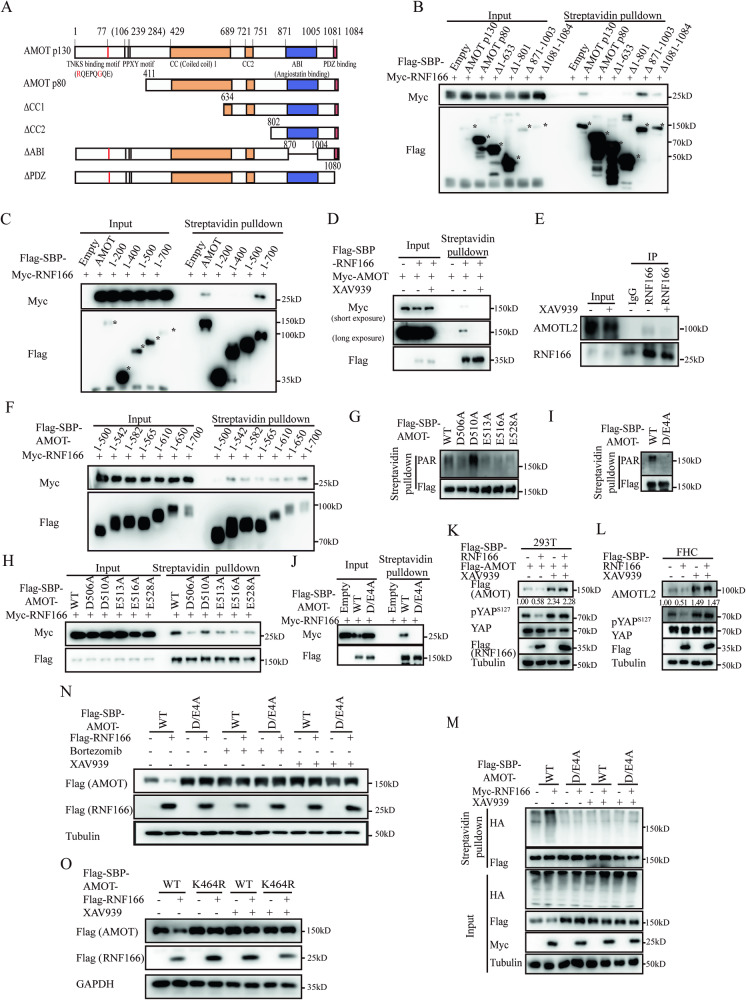
RNF166 regulates Motins through TNKS mediated PARsylation. **A** Schematic diagrams of WT and different truncated constructs of the AMOT. The key domains (rectangles) and motifs (vertical lines) are labeled above. **B** The N-terminal region (aa 1-411) of AMOT was required for AMOT binding to RNF166. The relevant plasmids were transfected into 293 T cells for 48 h. Interactions were detected by streptavidin pulldown, and different truncations of AMOT are labeled with asterisks. **C** AMOT aa 501–700 was essential for interaction with RNF166. **D** Effect of TNKS inhibitors on disrupting RNF166 binding to AMOT. After transiently transfecting 293 T cells with the indicated plasmids for 30 h, cells were then treated with DMSO or XAV939 (2 μM) for 18 h. These samples were further analyzed by streptavidin pulldown and the pulled proteins were immunoblotted. (**E**), XAV939 treatment affected the interaction between RNF166 and AMOTL2 in xenograft mode. **F** AMOT aa 1-542 mediated the interaction with RNF166. **G** Detection of PARsylation on AMOT WT and mutations at possible PARsylated sites. The experiment was performed as the ubiquitination assay but required the addition of PDD00017273 (5 μM) to the cell lysates. **H** The interaction between RNF166 and AMOT WT or PARsylated site mutations. D/E4A mutations abolished AMOT PARsylation (**I**) and prevented their binding to RNF166 (**J**). Effects of RNF166 and XAV939 on the stabilization of Motins in 293 T and FHC cells. 293 T cells transiently expressed Flag-AMOT and Flag-RNF166 (**K**), whereas FHC cells stably overexpressed Flag and SBP-tagged RNF166 (**L**). **M** Assay of ubiquitination of WT and D/E4A mutations of AMOT with DMSO or XAV939. Cells were transfected with the indicated plasmids, treated with DMSO or XAV939 (2 μM, 18 h) and prepared for the ubiquitination assay. **N** Effects of RNF166 overexpression, bortezomib, and XAV939 treatment on the stabilization of WT and D/E4A mutant AMOT. **O** Effect of RNF166 overexpression and XAV939 treatment on protein stabilization of WT and K464R AMOT. Abbreviations: A, alanine; aa, amino acids; ABI angiostatin binding domain, CC coiled coil; D/E4A, simultaneously mutated 4 sites (D506, E513, E516 and D528) to alanine (A) of AMOT; DMSO, dimethyl sulfoxide; PDD00017273, the inhibitor against poly (ADP ribose) glycohydrolase; WT, wild type.

To discover how RNF166 recognizes Motins, we aimed to identify the key domain responsible for the binding of AMOT to RNF166. Streptavidin pulldown experiments revealed that the N-terminal AMOTp130 significantly affected the recognition of AMOT by RNF166, as AMOTp80 (AMOTp130^∆1-410^) variant failed to pull down RNF166 (Fig. 4A, B). Surprisingly, truncations of AMOT from the C-terminus indicated that amino acids 501–700 were also essential for RNF166 binding (Fig. 4C). This suggests that there are two regions in AMOT required for binding to RNF166. We presumed that one region potentially acted as a regulatory element influencing the other region’s interaction with RNF166. The N-terminal AMOTp130 contains three PPxY motifs, a TNKS binding motif RxxxxGxx (R - arginine, G - glycine, x - any amino acid) and a highly conserved superhelix structure. These motifs are well conserved in all Motins except for the AMOTp80 variant (Fig. 4A). The TNKS binding motif allows AMOTp130 to conjugate to TNKS, which is a prerequisite for AMOTp130 PARsylation. Indeed, AMOTp130 and AMOT^1-200^, which contained the TNKS binding motif were capable of precipitating TNKS, while AMOTp80 was not (Fig. S6A). Recent studies have demonstrated that PARsylation directly mediates protein-protein interactions (PPIs) [9, 33, 34]. Intriguingly, several PARPs, including TNKS, were present in the PPI network of RNF166 (Fig. 2A). Thus, we hypothesized that AMOT was recognized by RNF166 only after being PARsylated by TNKS, and the region AMOTp130^501-700^ was the PARsylated region.

To test this hypothesis, we examined whether TNKS affects the interaction between RNF166 and Motins. Streptavidin pulldown revealed that the interactions between Motins and RNF166 were almost abolished by treatment with XAV939 (Figs. 4D and. S6B), an inhibitor of TNKS enzyme activity [33]. The interaction between RNF116 and AMOTL2 was also disappeared in XAV939-treated HCT116 cell xenograft tumors by immunoprecipitation (Fig. 4E). This indicated that TNKS activation was necessary for RNF166’s interaction with Motins. Next, we aimed to identify the specific PARsylated sites within AMOT responsible for the interaction with RNF166. More accurate truncations of AMOT from the N-terminus revealed that AMOTp130^1-542^ was the minimal truncation that mediated their interaction, suggesting that the key modification sites were located within amino acids 501–542 (Fig. 4F). As TNKS-induced PARsylation primarily occurs at aspartate (D) and glutamate (E) residues [35, 36], five conserved D and E residues were present within this region (Fig. S6C). Next, we mutated these amino acids into alanine residues (A) and tested their PARsylation separately. Four of the five mutations, AMOT D506A, E513A, E516A and E528A, were significantly attenuated PARsylation (Fig. 4G), and weakened their interactions with RNF166 (Fig. 4H). These findings indicated that these four sites were responsible for PARsylation. Simultaneous mutation of these four amino acids, termed them D/E4A hereafter, led to extremely low PARsylation levels and disappeared interaction with RNF166 (Fig. 4I, J). Similar results were observed for AMOTL2 D/E4A (Fig. S6D, E).

Having demonstrated that the interplay between Motins and RNF166 required TNKS-mediated PARsylation, we investigated whether Motins degradation caused by RNF166 was also regulated by TNKS. It was found that RNF166 overexpression no longer reduced Motins levels when treated with XAV939 in both 293 T and FHC cells (Fig. 4K, L). Moreover, the ubiquitination of Motins D/E4A was dramatically reduced compared to Motins WT and resistant to XAV939, regardless of the overexpression of RNF166 (Figs. 4M and S6F). Accordingly, both immunoblotting and immunofluorescence results showed that Motins D/E4A expression was significantly higher than that of WT, and this high expression was no longer affected by RNF166, with no response to XAV939 and bortezomib treatment (Figs. 4N, S6G, S5C). Finally, it was observed that XAV939 restored the reduction in WT Motins caused by RNF166 but had no effect on Motins with mutations in the ubiquitination sites (Fig. 4O). These above experiments reveal that TNKS mediates RNF166-induced destabilization of Motins.

### The c-terminus of RNF166 is a PBD responsible for PARsylation

**Fig. 5:**
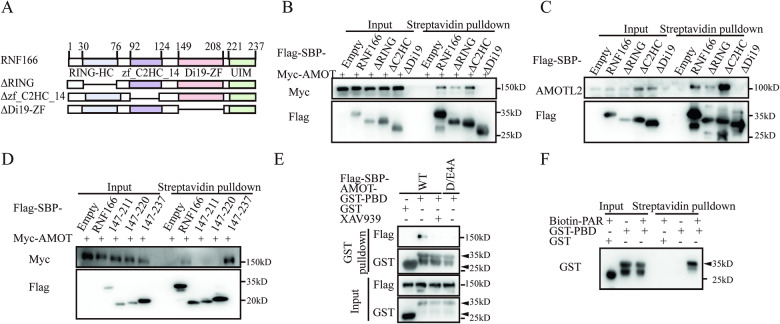
Recognition of PARsylation requires the C-terminal RNF166. **A** Schematic representation of WT and deletion mutants of RNF166. **B**, **C** The Di19-ZF domain is important for RNF166 to interact with Motins. **D** AMOT only binds to WT RNF166 and C-terminal RNF166, instead of other truncated RNF166. **E** Only AMOT, but not XAV939 pretreated AMOT or AMOT D/E4A could be pulled down by GST-PBD. GST-fused proteins were purified from E. coli and incubated with Flag and SBP-tagged AMOT from 293 T cells. XAV939 was added to the cell and pulldown buffer in the XAV939 treatment group. **F** Streptavidin pulldown to analyze the PARsylation binding activity of GST-PBD. The position of GST-PBD is marked with a black triangle. Abbreviations: GST glutathione *S*-transferase, PBD PAR-binding domain, ZF zinc finger.

To understand how RNF166 recognized the PARsylated Motins, we examined different domains of RNF166 and their interactions with Motins. Deleting the ZF-Di19 domain of RNF166 abolished the interaction between RNF166 and Motins, indicating the importance of this domain (Fig. 5A, C). Surprisingly, the Di19-ZF domain itself could not directly bind to AMOT. However, when the Di19-ZF domain was extended to the C-terminus, which contained a UIM motif, the entire C-terminus was found to the key region for binding to AMOT (Fig. 5D).

Considering that RNF166 recognized PARsylated Motins, it was hypothesized that the C-terminus of RNF166 functions as a PBD. To test this hypothesis, we purified the C-terminus of RNF166 fused with glutathione *S*-transferase (GST-PBD). GST pulldown assay confirmed that purified GST-PBD directly interacted with Motins but not with Motins treated with XAV939-treated or mutated in the D/E4A sites (Fig. 5E). Notably, an in vitro streptavidin pulldown assay using biotin-labeled poly ADP-ribose polymer demonstrated that GST-PBD was detected in the precipitated fraction (Fig. 5F), providing direct evidence of its recognition of PARsylation. The C-terminus of RNF166, including the Di19-ZF domain, structurally differs from previously reported PBDs such as WWE [37], PAR-binding zinc-finger domain [38], microdomain [39], ring [40] and WD40 domains [41]. Therefore, the C-terminus of RNF166 represents a novel PBD responsible for the recognition of PARsylated Motins.

### RNF166 regulates the Hippo pathway through Motins

**Fig. 6:**
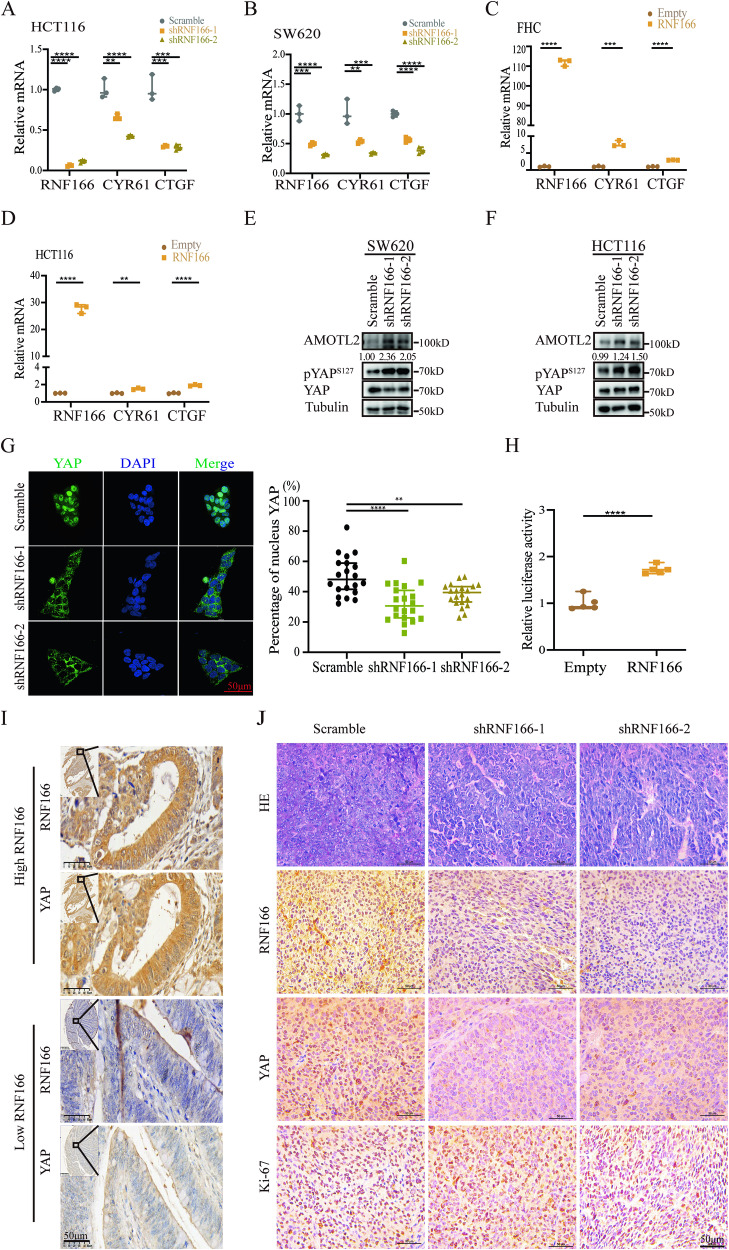
RNF166 promotes CRC progression by regulating the Hippo pathway. **A**, **B** YAP target genes CTGF and CYR61 were detected by qPCR in stable RNF166-depleted and scramble CRC cells. **C**, **D** mRNA expression of CTGF and CYR61 was analyzed in FHC and HCT116 cells stably overexpressing RNF166. **E**, **F** AMOTL2 increasement and YAP inactivation were observed in CRC cells with stable knockdown of RNF166 by immunoblotting. **G** Immunofluorescence analysis and quantitative analysis of the YAP distribution in HCT116 cells with stable knockdown of RNF166. **H** Luciferase activity was detected in the 293 T cells by transfecting RNF166 or empty vector plasmids and luciferase reporter. **I** IHC of serial sections showed consistent expression of RNF166 and YAP in human CRC tissues and tumors. Scale bar, 50 μm. **J** H&E and IHC analysis of RNF166, YAP and Ki-67 levels in scramble or RNF166-depleted xenograft tumors. Scale bar, 50 μm. For (**A**–**D**) and (**H**), **p < 0.01, ***p < 0.001, and ****p < 0.0001.

Above, we demonstrated that RNF166 degraded Motins, which are regulatory components of the Hippo pathway. Then, we further investigated the association between RNF166 and the Hippo pathway. Positive correlations were observed between the mRNA levels of RNF166 and YAP target genes in COAD tissues from cBioPortal. (Fig. S7A, B) [42]. Knockdown of RNF166 in CRC cells resulted in significantly lower mRNA levels of CYR61 and CTGF (Fig. 6A-B). Conversely, the mRNA levels of these genes were elevated in FHC and HCT116 cells after overexpressing RNF166 (Fig. 6C, D). We also examined the effect of RNF166 on YAP phosphorylation at serine 127 (pYAPS127). pYAPS127 increased after RNF166 knockdown and decreased after RNF166 overexpression in HCT116 and SW620 cells (Figs. 6E, F, 4K, L, and Fig. S7C–E). IF showed significant cytoplasmic retention of YAP in RNF166 knockdown cells (Fig. 6G). Reporter gene assay further supported that RNF166 overexpression increased the transcriptional activity of YAP (Fig. 6H). Moreover, IHC analysis of COAD and adjacent tissues showed that high expression of RNF166 was accompanied by elevated YAP and nuclear localization (Fig. 6I). Consistently, reduced RNF166 in xenograft tumors correlated with decreased YAP intensity and Ki-67-positive cells by IHC (Fig.6J), and increased AMOTL2, pYAPS127 by immunoblotting (Fig. S7F). In addition, YAP activation induced by RNF166 overexpression was abrogated by XAV939 (Fig. 4K, L). XAV939 also affected the size of xenograft tumors and increased levels of AMOTL2 and phosphorylated YAP in vivo (Fig. S7G, H). Taken together, these results collectively indicated that RNF166 negatively regulated the Hippo pathway and enhanced YAP activation.

**Fig. 7:**
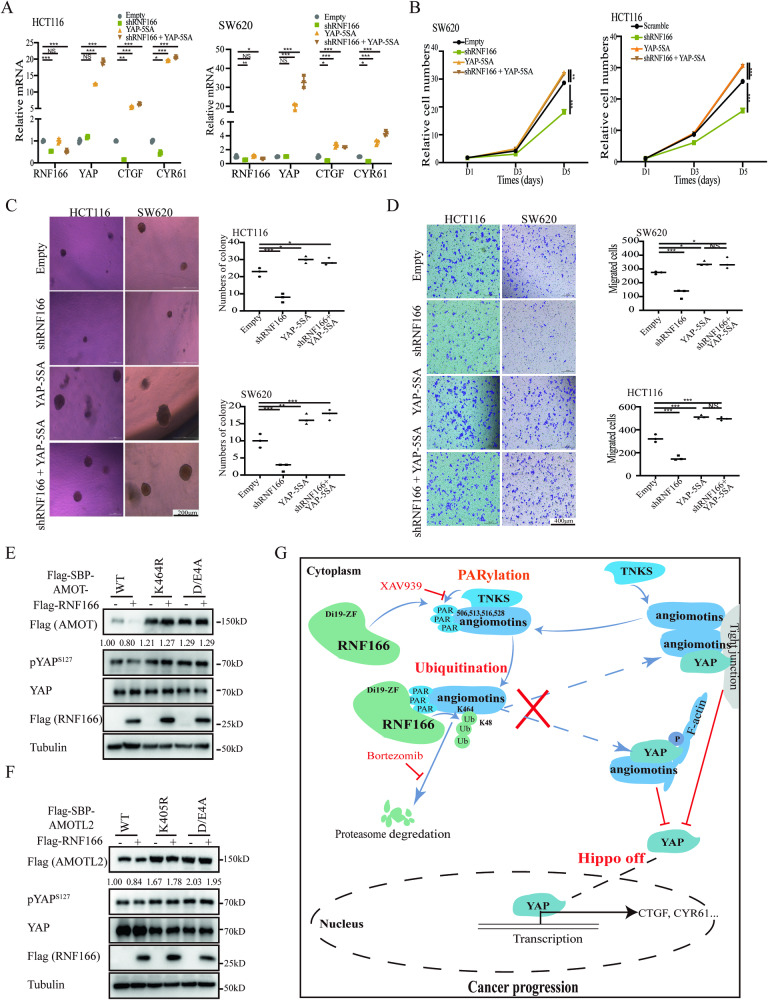
YAP-5SA rescues the knockdown of RNF166-mediated Hippo on. The control or RNF166 knockdown SW620 and HCT116 cells were also stably transfected with empty vector or activated YAP-5SA. Overexpression of YAP-5SA rescued RNF166 KD-induced YAP target gene downregulation (**A**). Meanwhile, YAP-5SA OE also rescued RNF166 KD-induced proliferation deficits (**B**), anchorage-independent growth inhibition (**C**), and cell mobility attenuation (**D**) in CRC cells. **E**, **F** Mutations of ubiquitination or PARsylation sites of Motins in 293 T cells effectively abolished RNF166 OE-induced YAP activation. **G** Graphic illustration showing the role of RNF166 in CRC tumorigenesis. In the progression of CRC, TNKS recognizes PARsylated Motins (at D506, E513, E516 and D528 of AMOT), which are recognized by the C-terminus of RNF166. RNF166 bound PARsylated Motins and then induced K48-linked polyubiquitination modification (at K464 of AMOT), thereby mediating their proteasomal degradation. Degraded Motins led to increased dephosphorylation and elevated YAP accumulation in the nucleus (also referred to Hippo inactivation), which ultimately contributed to tumor progression. Data are presented as the mean ± SD. Abbreviations: DAPI, 2-(4-Amidinophenyl)-6-indolecarbamidine dihydrochloride; KD, knockdown; NS, not significant; OE, overexpression; pYAP^S127^, phosphorylated YAP at serine 127; qPCR, real-time PCR assay. For (**A**–**D**), *p < 0.05; **p < 0.01, and ***p < 0.001.

Having demonstrated the role of RNF166 in promoting YAP transcriptional activity, we then investigated whether RNF166 regulated COAD progression through YAP. The introduction of activated YAP (YAP-5SA) [43] significantly increased target gene expression (Fig. S1C, D, and Fig. 7A) and improved cell proliferation (Fig. 7B), anchorage-independent growth (Fig. 7C), and cell migration (Fig. 7D), while RNF166 knockdown did not further reduce tumor cell malignancy following YAP-5SA expression. Therefore, the regulation of YAP activation promotes COAD progression.

Next, we investigated whether RNF166 regulated the Hippo pathway and promoted tumor progression by degrading Motins. Both mutations in the ubiquitination and PARsylation sites of Motins abrogated the Hippo inactivation caused by RNF166 overexpression (Figs. 7E, F, S7I). Depletion of RNF166 in CRC cells reduced YAP activation, and this reduction was fully rescued by AMOTL2 knockdown (Fig. S1E, F). Taken together, RNF166 mediates CRC progression by regulating the Hippo pathway via the TNKS-RNF166-Motins axis.

## Discussion

YAP, the core of the Hippo pathway, is involved in the homeostasis and stemness of the intestinal epithelium. Hyperactive YAP is associated with colorectal cancer tumorigenesis and drug resistance [4]. While the scaffold and polarity proteins involved in Hippo pathway regulation are well-defined, the regulation of Motins remains to be elucidated. Herein, we discovered that overexpressed RNF166 directly targeted the Motins and cliented them to the proteosome in response to PARsylation, resulting in activation of YAP (Fig. 7G). The activation effect of RNF166 on YAP was abrogated by the TNKS inhibitor XAV939, while the deprived function resulting from RNF166 knockdown was rescued by YAP-5SA overexpression or AMOTL2 knockdown. RNF166 recognized PARsylation by TNKS mainly through the C-terminal PBD, which differed from the previous PBD. In addition, we found that RNF166 was a prognostic biomarker of CRC. This work sheds light on upregulated RNF166 acts as a positive regulator of the oncogene YAP through the TNKS-RNF166-Hippo axis, providing a potential therapeutic target for CRC treatment and a prognostic biomarker for CRC patients.

Previous studies have reported the involvement of RNF166 in inflammation, autophagy, and immunity, but its role in cancer is unclear. Here, we demonstrated RNF166 as a tumor promoter in CRC from clinical cohort data, the malignant function of tumor cells in vitro, and tumorigenesis in vivo after RNF166 knockdown. Importantly, we discovered a novel function of RNF166 as a reader of PARsylation. Recognition of Motins by RNF166 required the TNKS binding motif. Moreover, XAV939 was effective in inhibiting RNF166 binding to Motins, degrading Motins, and regulating the Hippo pathway, demonstrating that RNF166 recognized PARsylated Motins. It is worth noting that the isoform AMOT-p80 lacks the N-terminal region, which not only contains the PPxY motifs that bind to YAP but also covers the TNKS binding motif [44]. This structural difference may explain its insensitivity to TNKS regulation and recognition by RNF166. In addition, our study identified the PARsylation sites of Motins by TNKS. Another E3 ligase, RNF146, was previously reported as a PARsylation-directed E3 ubiquitin ligase through its WWE domain [45]. RNF146 also promotes YAP activity by degrading Motins [9]. RNF146 is involved in TNF-induced cell death [46], anti-DNA virus immunity via VISA [47], and the WNT pathway [45, 48], all through TNKS. The reported involvements of RNF146 coincidentally overlapped with predicted function by enrichment and previously reported function of RNF166, because RNF166 interacted with several immune-related proteins, e.g., VISA and TRAF3/6, and PARPs, e.g., PARP1, PARP11, and PARP12, suggesting that RNF166 may also function as a PARsylation-directed E3 ubiquitin ligase. Although our study did not further validate this hypothesis or explore the diverse roles of RNF166 in tumors, it provides valuable insights into the potential functions of RNF166 in various cellular processes.

By conducting pulldown assays with domain-deleted forms of RNF166 and Motins, the C-terminal region of RNF166, which contains the Di-ZF domain, plays a critical role in Motin recognition. Importantly, biotin-labeled poly ADP-ribose polymer directly pulled down purified GST-PBD in vitro, indicating that the C-terminus was the right PBD. PBDs read PARsylation by positively charged amino acids (lysine, arginine, and histidine) binding to negatively charged ADP molecule [41]. We found that the region responsible for PARsylation recognition was a cluster of five intensively positively charged amino acids, amino acids 203–209 (HLLHRHK), located at the end of the Di19-ZF domain. To our surprise, the domain containing only Di19-ZF (amino acids 149–208) did not directly recognize PARsylation but did require the entire C-terminus, even though no positively charged amino acids were present within amino acids 209–237. Similarly, the Di19-ZF domain is also conserved in other E3 ligases such as RNF125, RNF138, RNF114, and KCMF1; thus, they may also participate in PARsylation-directed protein homeostasis. Further biochemistry and structural knowledge on RNF166 are warranted to elucidate this PARsylation-PBD complex and to design small molecule inhibitors or peptides modulating in PARsylation-PBD interactions.

## Materials and methods

### Antibodies and reagents

The following primary antibodies were used in Western blots (WB) : anti-RNF166 (Abcepta AP20484c, 1/1000), anti-AMOT (Cell Signaling Technology 43130, 1/1000), anti-AMOTL2 (Thermo PA5-78770, 1/2500), anti-YAP1 (ZENBIO 380482, 1/2 500), anti-Phospho-YAP (Ser127) (ZENBIO 310307, 1/2500), anti-Beta Actin (Proteintech 81115-1-RR, 1/5000), anti-β-tubulin (Abmart M20005, 1/5000), anti-GAPDH (Goodhere AB-P-R001, 1/3000), anti-Poly(ADP-Ribose) polymer (Abcam ab14459, 1/1000), anti-HA (Abmart M20003, 1/5000), anti-GST (Abmart M20007, 1/5000), anti-Myc (Abmart M20002, 1/5000) and anti-Flag (Abmart M20008, 1/5000; and CST 14793, 1/5000) antibodies. Secondary antibodies were purchased from APExBIO (K1221 and K1223, 1/5000) and Abbkine (A25022 and A25222, 1/2000).

Key reagents were purchased: Biotin (terminal)-poly ADP-ribose (PAR) polymer (Trevigen, 4336-100-02), XAV939 (Selleck, S1180), Bortzeomib (Beyotime, SC0263), PDD00017273 (GlpBio, GC32784), Blasticidin S HCl (Beyotime, ST018), Puromycin (Solarbio, P8230), Streptavidin Agarose Resin 6FF (Yeasen, 20512ES08), and Glutathione Sepharose (GE Healthcare, 17-0756-01).

### Cell culture

293 T cells were obtained from National Collection of Authenticated Cell Cultures, SW620 and FHC cells were purchased from ZQXZbio, HCT116 cells were a kind gift from Ya Cao, and HCT-8 were a kind gift from Kai Fu. SW620, HCT116 and FHC cells were cultured in RPMI Medium 1640 basic (Gibco, C11875500BT), while 293 T and HCT-8 cells were grown in DMEM basic (Gibco, C11995500BT). All media were supplemented with 10% fetal bovine serum premium (NEWZERUM, FBS-CP500) and 1% 100× penicillin–streptomycin solution (Coolaber, SL6040) in a 5% CO_2_ incubator at 37 °C.

### Construction of plasmids and mutagenesis

Human RNF166, AMOT and AMOTL2 expression plasmids in the pENTER vector were purchased from Vigene Biosciences (CH893837, CH808629 and CH821284), and pCMV-flag YAP2-5SA [43] was a gift from Kunliang Guan (Addgene plasmid #27371). The human TNKS plasmid was generated by PCR amplification of the corresponding cDNAs and cloning into pcDNA 3.1. The indicated plasmids were subcloned into pLV-EF1α-MCS-IRES-Puro, pLV-EF1α-MCS-IRES-Bsd, pcDNA 3.1 or pGEx-6P1 expression vectors with various tags, depending on the experimental purposes. The lentiviral packaging plasmids pMD2.G (#12259) and psPAX2 (#12260) and ubiquitin-related plasmids pRK5-HA-Ubiquitin-WT (#17608), pRK5-HA-Ubiquitin-K48 (#17605), and pRK5-HA-Ubiquitin-K63 (#17606) were obtained from Addgene.

RNF166 shRNA was generated according to the Addgene’s pLKO.1-TRC cloning vector protocol. The targeting sequences of RNF166 were as follows: #1 sense: 5′-GAAGCAGCTCTCATCCTACAA-3′ and #2 sense: 5′-CGTCTCTTCAAAGCCATGATA-3′.

### Transfection and construction of lentivirus-infected stable cells

For 293 T cells, plasmids were transfected with polyethyleneimine (Yeasen, 40816ES02) according to the manufacturer’s protocol. To generate stable RNF166 knockdown/overexpression and/or YAP-5SA overexpression cells, the indicated plasmids were cotransfected with lentiviral packaging plasmids for 48 h in 293 T cells to generate lentivirus. Cells were screened by 2 μg/mL puromycin and/or 10 μg/mL Blasticidin S.

AMOTL2 knockdown in colorectal cancer cells was performed by jetPRIME (Polyplus, 101000046). The siRNA sequences targeting AMOTL2 were as follows: #1 sense: 5′-CTCATGTTGTACTAGCTCA-3′ and #2 sense: 5′-CAGTGATCAAGGTCCTTCA-3′ (Tsingke).

### Western blotting

Samples were lysed with WB lysis buffer (50 mM Tris-HCL, pH 7.5, 150 mM NaCl, 1% Triton-X) containing protease inhibitor cocktail (APExBIO K1007, 1/100) and phosphatase inhibitor cocktail (APExBIO K1014, 1/100). Lysates were centrifuged at 15,000 g at 4 °C for 15 min, and the supernatants were denatured with Laemmli SDS sample buffer, reducing (6×) (Thermo, J61337) at 100 °C for 15 min. The final samples were loaded onto 6–12% SDS–PAGE gels according to the molecular weight of the indicated proteins, transferred to PVDF membranes (Millipore), blocked with 3% bovine serum albumin, and immunoblotted with the indicated antibodies. The immunoblotting results were repeated at least three times.

### Co-immunoprecipitation and pull-down assay

Cells were lysed on ice with IP lysis buffer (50 mM Tris-HCL, pH 7.5, 150 mM NaCl, 0.5% NP-40) containing a protease inhibitor cocktail. Supernatants were obtained after centrifugation and then incubated with streptavidin agarose. The precipitates were washed with IP lysis buffer, boiled with SDS sample buffer, and analyzed by Western blotting.

For GST pulldown, GST and GST-PBD recombinant proteins were expressed in Rosetta (DE3) cells and induced by treatment with 0.5 mM isopropyl-β-D-thiogalactopyranoside at 37 °C for 3 h. GST recombinant proteins were purified with Glutathione Sepharose. Flag-SBP-AMOT was expressed in 293 T cells and purified with streptavidin agarose. When ready for GST pulldown, Glutathione Sepharose was incubated with recombinant protein and Flag-SBP-AMOT mixture on a rotor at 4 °C for 3 h. The precipitates were washed, denatured with 2× SDS sample buffer, and finally detected by SDS–PAGE gels.

For the PAR binding assay, 0.4 μmol GST or GST-PBD recombinant protein was incubated with 1 μL 100 μM biotin (terminal)-PAR polymer and 10 μL streptavidin agarose in a total volume of 400 μL IP lysis buffer. Proteins bound to PAR polymer could be precipitated by streptavidin agarose and detected by anti-GST antibody for WB.

### Ubiquitination assay and PARsylation assay

Ubiquitination and PARsylation modifications were tested in 293 T cells transfected with the indicated plasmids. Cells were initially lysed with strong lysis buffer (50 mM Tris-HCL, pH 7.5, 150 mM NaCl, 1% Triton-X, 1% SDS) and then diluted to 0.1% SDS with 9-fold WB lysis buffer. Target proteins were precipitated by streptavidin agarose. The additional PARG inhibitor PDD00017273 was added to the lysis buffer when assessing PARsylation. Detection of final samples was performed with 6–8% SDS–PAGE and the indicated antibodies.

### RNA extraction and quantitative real-time PCR assay

RNA was extracted with total RNA extraction reagent (Vazyme, R401-01) reverse transcribed with HiScript III RT SuperMix (Vazyme, R323-01), and real-time PCR was completed with ChamQ Universal SYBR qPCR Master Mix (Vazyme, Q711-02). Expression of human GAPDH or β-actin was used for normalization. The primers used were RNF166 (F: 5′-CTATGTTCCGCAGCCTGGT-3′; R: 5′-CAGATGGGGCAGGTGTACTG-3′); CYR61 (F: 5′-CAGGACTGTGAAGATGCGGT-3′; R: 5′-GCCTGTAGAAGGGAAACGCT-3′); CTGF (F: 5′-GTTTGGCCCAGACCCAACTA-3′; R: 5′-GGCTCTGCTTCTCTAGCCTG-3′); AMOT (F: 5′-GGAGTTAAGGCCCATCCACC-3′; R: 5′-GCCCTTGGGCCTTGTAGAAT-3′) AMOTL2 (F: 5′-ATCCTAGAGGAGGGGTGTCC-3′; R: 5′-TGCAGAAGCCGTTCACTCAA-3′); YAP1 (F: 5′-TTCAACGCCGTCATGAACCC-3′; R: 5′-GCTTGAAGAAGGAGTCGGGC-3′); GAPDH (F: 5′-GAAACTGTGGCGTGATGGC-3′; R: 5′-CCGTTCAGCTCAGGGATGAC-3′) and β-actin (F: 5′-CTTCGCGGGCGACGAT-3′; R: 5′-CCACATAGGAATCCTTCT-3′).

### Immunofluorescence microscopy

Cells were cultured on glass plates, fixed with 4% paraformaldehyde solution, permeabilized with 0.5% Triton X-100, blocked with 3% bovine serum albumin, and probed with the indicated antibodies (mouse anti-Flag antibody, dilution 1/200; rabbit anti-Flag antibody, dilution 1/200; mouse anti-Myc antibody, dilution 1/200; rabbit anti-YAP1 antibody, dilution 1/100; rabbit anti-AMOT antibody, dilution 1/50, and rabbit anti-AMOTL2 antibody, dilution 1/100) at 4 °C overnight. Samples were then incubated with secondary antibodies (Abbkine, A23310 and/or A23220, dilution 1/150) and stained with DAPI (Solarbio, C0065). Finally, these images were collected under a NIKON microscope. Quantitative analysis of colocalization or fluorescence intensity was performed with Image J (https://imagej.net/ij/) and the Z-axis information was analyzed using the NIS Elements viewer (https://www.microscope.healthcare.nikon.com/zh_CN/).

### Immunohistochemistry

Colon cancer tissue microarrays containing 75 cases with cancer and adjacent normal tissues were provided by OUTDO BIOTECH (HColA150CS02; https://www.superchip.com.cn/) and were approved by the ethical committee (No. SHXC2021YF01). Two cases were excluded due to tissue loss. Antigen retrieval was performed with sodium citrate buffer, and immunostaining was performed according to the instructions of immunohistochemistry kits (ZSGB-BIIO, PV-9000 and ZLV-9018). The antibodies used for IHC were anti-RNF166 (dilution 1/100), anti-YAP (dilution 1/100), and anti-KI67 (Proteintech 27309-1-AP, dilution 1/100).

### Dual luciferase reporter gene assay

For the dual luciferase reporter gene assay, pENTER-RNF166 or pENTER plasmids were cotransfected with pRL-TK and 8xGTIIC-luciferase plasmids (Addgene #34615) into 293 T cells. The relative light units of each group were measured according to the instructions of the Dual Luciferase Reporter Gene Assay Kit (Beyotime, RG027).

### CCK8 assay, transwell assay and anchorage-independent growth assay

Cell proliferation viability was assessed using the CCK8 assay. Cells (2 × 10^3^ cells/well) were seeded into 96-well plates, and cell viabilities were measured at a wavelength of 450 nm after incubation with fresh medium containing 10% cytotoxicity kit-8 solution (Vazyme, A311-01) for 30 min at 37 °C.

For transwell experiments, SW620 and HCT116 cells were washed and suspending with serum-free RPMI Medium 1640. Cells (2 × 10^4^ cells/well) were seeded on the upper chambers (8.0 μM pore size, Corning, 3422), which were placed on a 24-well plate containing 650 μL of serum-1640 medium. After 30 h of incubation, cells that migrated to the other surface of the chamber bottom were collected. The migrated cells were stained with crystal violet, photographed, and counted after fixation with 4% paraformaldehyde.

Anchorage-independent growth was performed in a 24-well plate injected with two layers of medium containing low melting point agar. The bottom layer consisted of 400 μL complete DMEM containing 0.6% soft agar, followed by the upper layer containing 1000 cells suspended in 400 μL complete DMEM containing 0.35% soft agar. After 2 weeks of incubation, the number of colonies in each group was counted.

### Xenograft tumorigenesis study

The xenograft study was conducted according to the guidelines approved by the ethical committee of Hunan SJA Laboratory Animal Co., Ltd. (SJA2023078-3) and the ethical committee of the Ethical Committee of Xiangya Hospital (202309030). Fifteen male BALB/c nude mice (5 weeks old) were randomly allocated into three groups (five mice in each group). Approximately 9 × 10^6^ HCT116 cells mixed with 60 μL PBS and 60 μL Matrigel Matrix (Corning, 356234) were subcutaneously inoculated into each mouse. For xenograft tumorigenesis treated with XAV939, 5 × 10^6^ HCT116 cells were inoculated of 10 male BALB/c nude mice. Scramble cells were inoculated on the left and RNF166 knockdown cells were inoculated on the right. XAV939 (20 mg/kg) or DMSO was dosed every 3 days by intraperitoneal injections on the 5th day after inoculation. The tumor volumes were calculated with the following formula: V = 1/2 × long diameter × square of short diameter.

### Statistical analysis

GraphPad Prism software (Version 9) was used for statistical analysis. Quantitative data were processed with two-tailed Student’s *t* test for two groups or ANOVA for multiple groups. Descriptive data were analyzed with the χ² test. In the figures, statistical differences are denoted as NS for no significance, * for *p* < 0.05, ** for *p* < 0.01, *** for *p* < 0.001, and **** for *p* < 0.0001.

### Supplementary information

Supplementary figures and table

checklist

Original Data File

## Data Availability

The datasets used and analyzed during the current study are included within the article and available from the corresponding authors upon reasonable request.
