# Supplementary material for: RNF166 promotes colorectal cancer progression by recognizing and destabilizing poly-ADP-ribosylated angiomotins
Source: Cell Death Dis. 2024 Mar 13;15(3):211. doi: 10.1038/s41419-024-06595-9 (PMC10937711; doi:10.1038/s41419-024-06595-9)
Supplement: Supplementary file 1 — Supplementary figures and table [file 41419_2024_6595_MOESM1_ESM.docx]

**Supplementary figures**


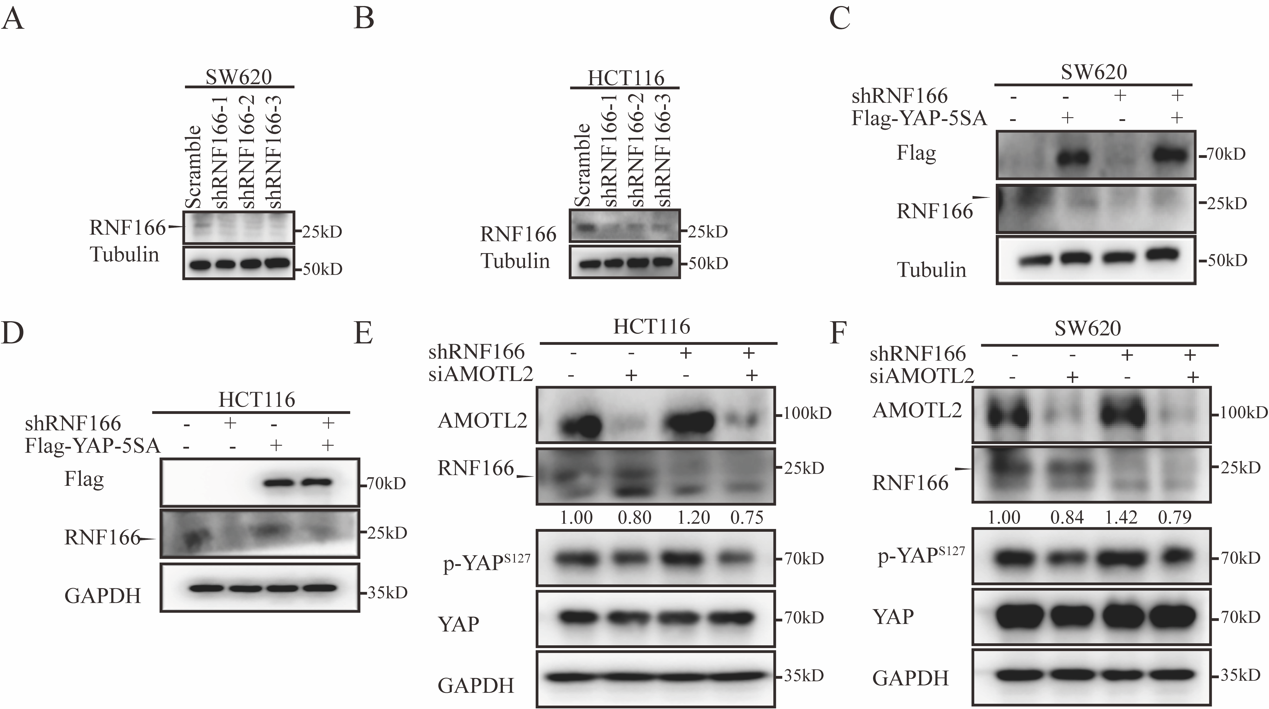


**Fig. S1: Effects of gene knockdown or overexpression by immunoblots in indicated cells.**

Validation of stable depletion of RNF166 by shRNA in SW620 cells (A) and HCT116 cells (B). YAP-5SA was stably transfected into scramble or RNF166 knockdown SW620 (C) and HCT116 (D) cells. AMOTL2 knockdown by siRNA in CRC cells with stable depletion of RNF166 was assessed by WB (E, F).


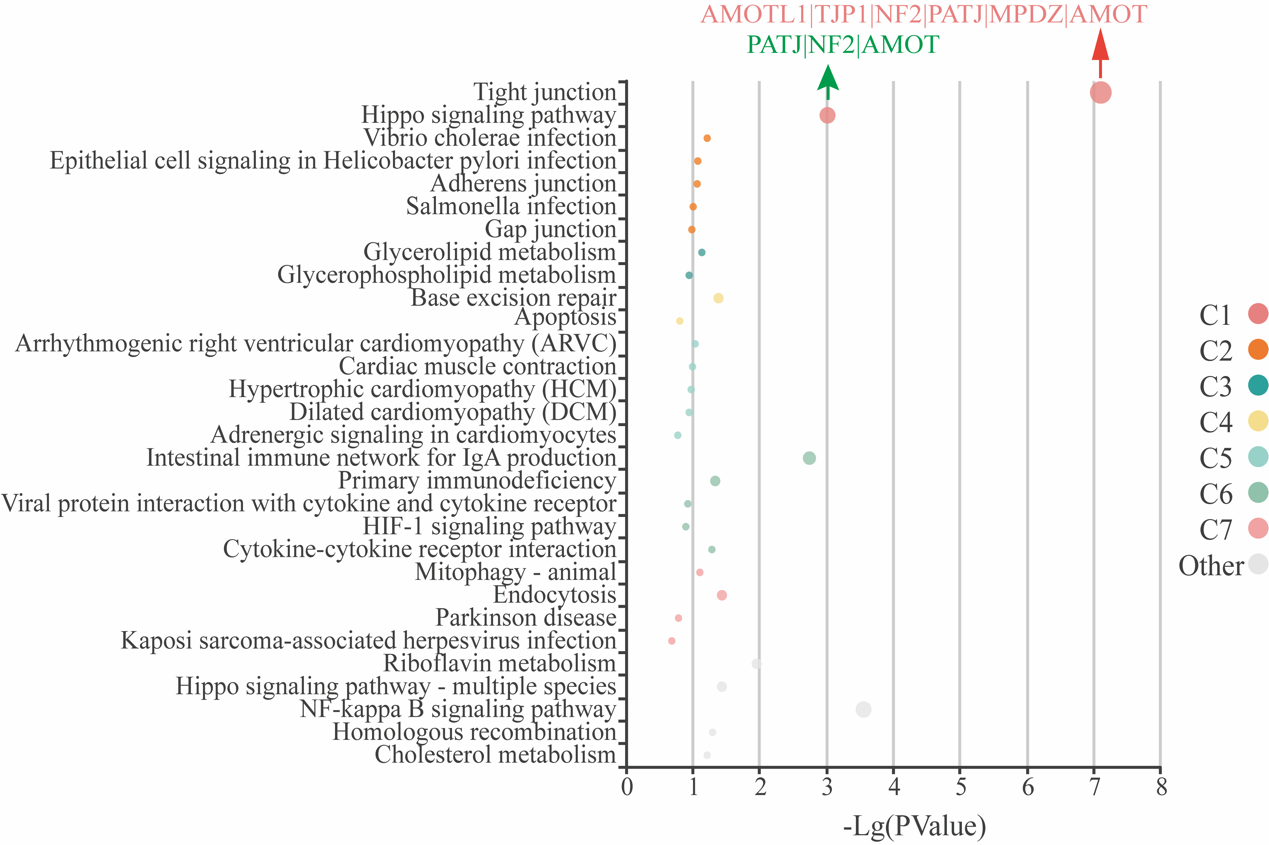


**Fig. S2: Enrichment analysis of the interacting proteins with RNF166 by KOBAS.** The key proteins involved in tight junction and the Hippo pathway are listed above.


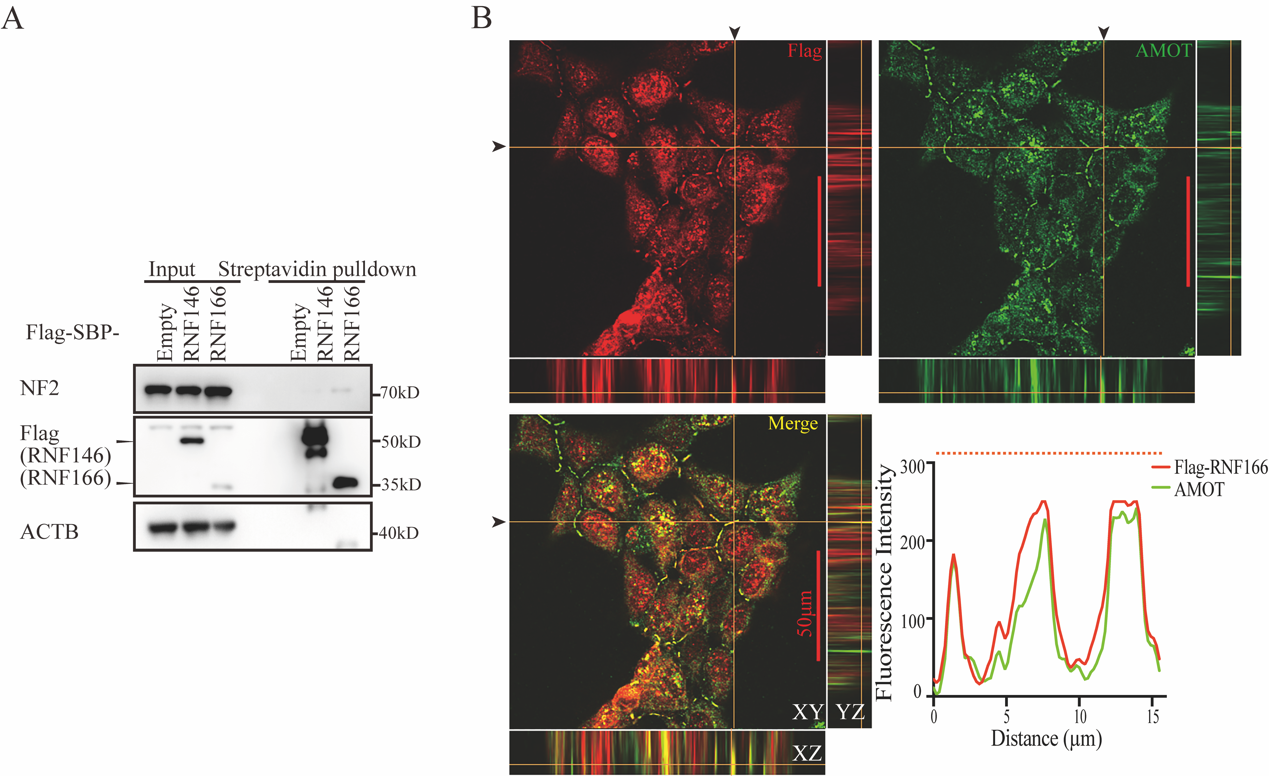


**Fig. S3: NF2 and AMOT are the interacting proteins of RNF166.**

(A) NF2 interacted with RNF166, and their interaction was stronger than that between NF2 and RNF146. (B) IF showed the colocalization of RNF166 (red) and AMOT (green) in HCT116 cells. Indicated (orange) intensity trace was plotted. HCT116 cells were stably overexpressed Flag-RNF166. Samples were coimmunized with mouse anti-Flag and rabbit anti-AMOT antibodies. Scale bar, 50 μm.


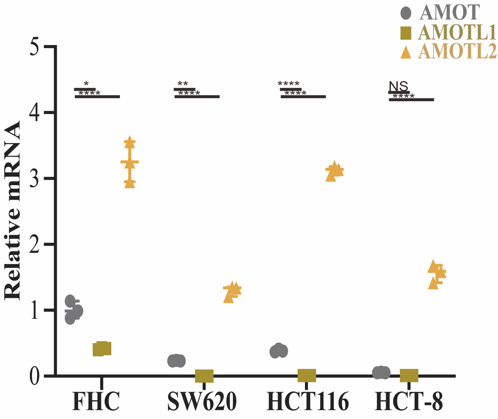


**Fig. S4: mRNA expression of Motins in FHC and CRC cell lines.** Target gene expression levels were corrected with the reference GAPDH and AMOT in FHC cells. *, *p* < 0.05; **, *p* < 0.01; ***, *p* < 0.001; and **** *p* < 0.0001.


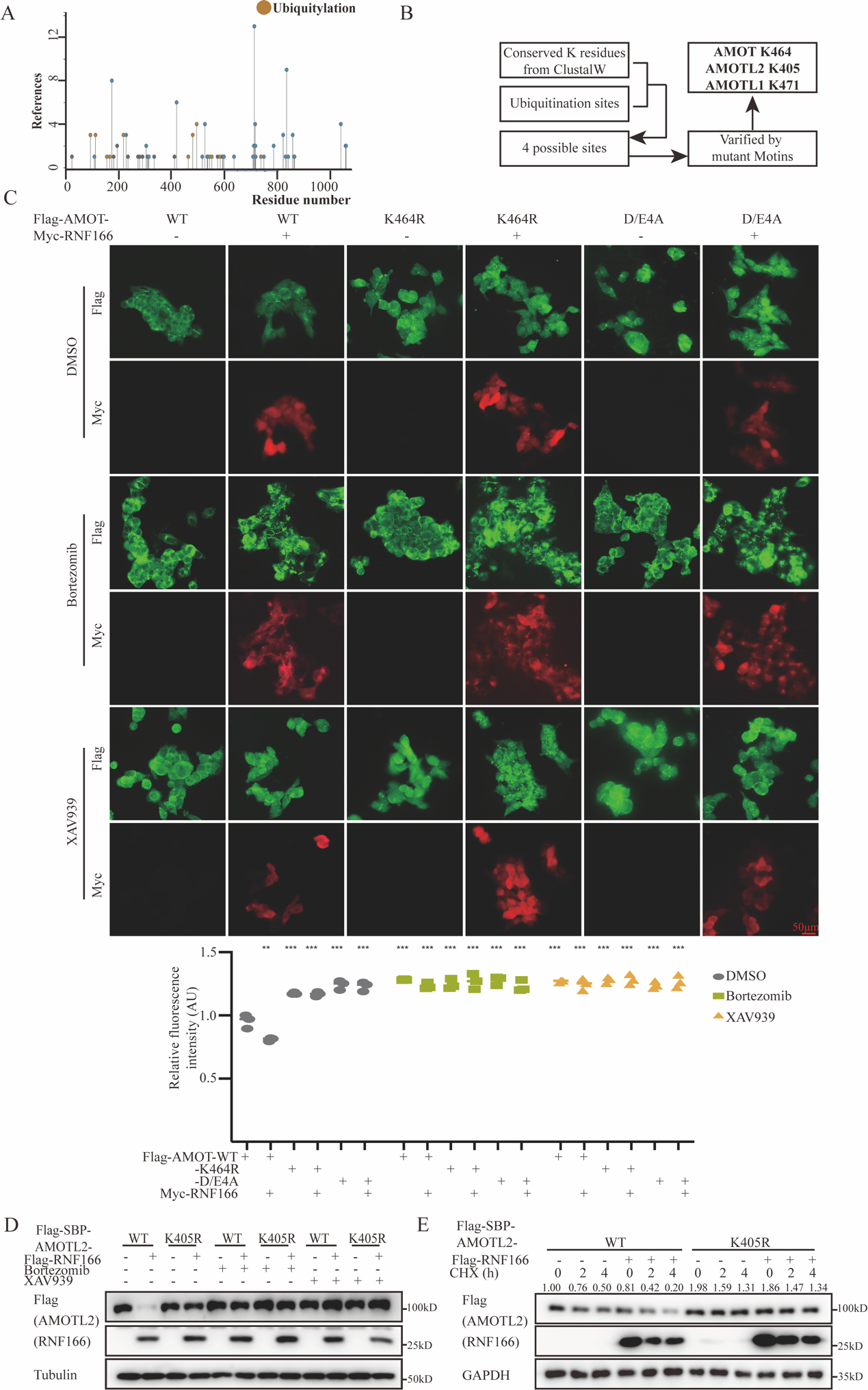


**Fig. S5: RNF166 degrades Motins.**

(A) The ubiquitination sites (brown dots) of AMOT from the PhosphoSitePlus database. A total of 11 sites of ubiquitination were identified: K94, K113, K156, K167, K219, K464, K484, K496, K545, K553 and K585. The horizontal axis represents the amino acid sites, while the vertical axis indicates the frequency of the indicated site from publications. (B) The flow diagram describes the screening of ubiquitination sites of Motins. (C) Immunofluorescence detects the relative abundance of AMOT wild-type, K464R, and D/E4A after overexpression of RNF166, bortezomib or XAV939 treatment in HCT116 cells. Mean immunofluorescence intensity is calculated as the integrated density within a certain area and is plotted below. (D) Mutation of AMOTL2 K405R is resistant to RNF166-induced degradation and insensitive to bortezomib and XAV939. (E) The AMOTL2 K405R mutation shows increased protein stability compared to wild-type AMOTL2. Abbreviations: AU, arbitrary units. ***, *p* < 0.001; and **** *p* < 0.0001.

**
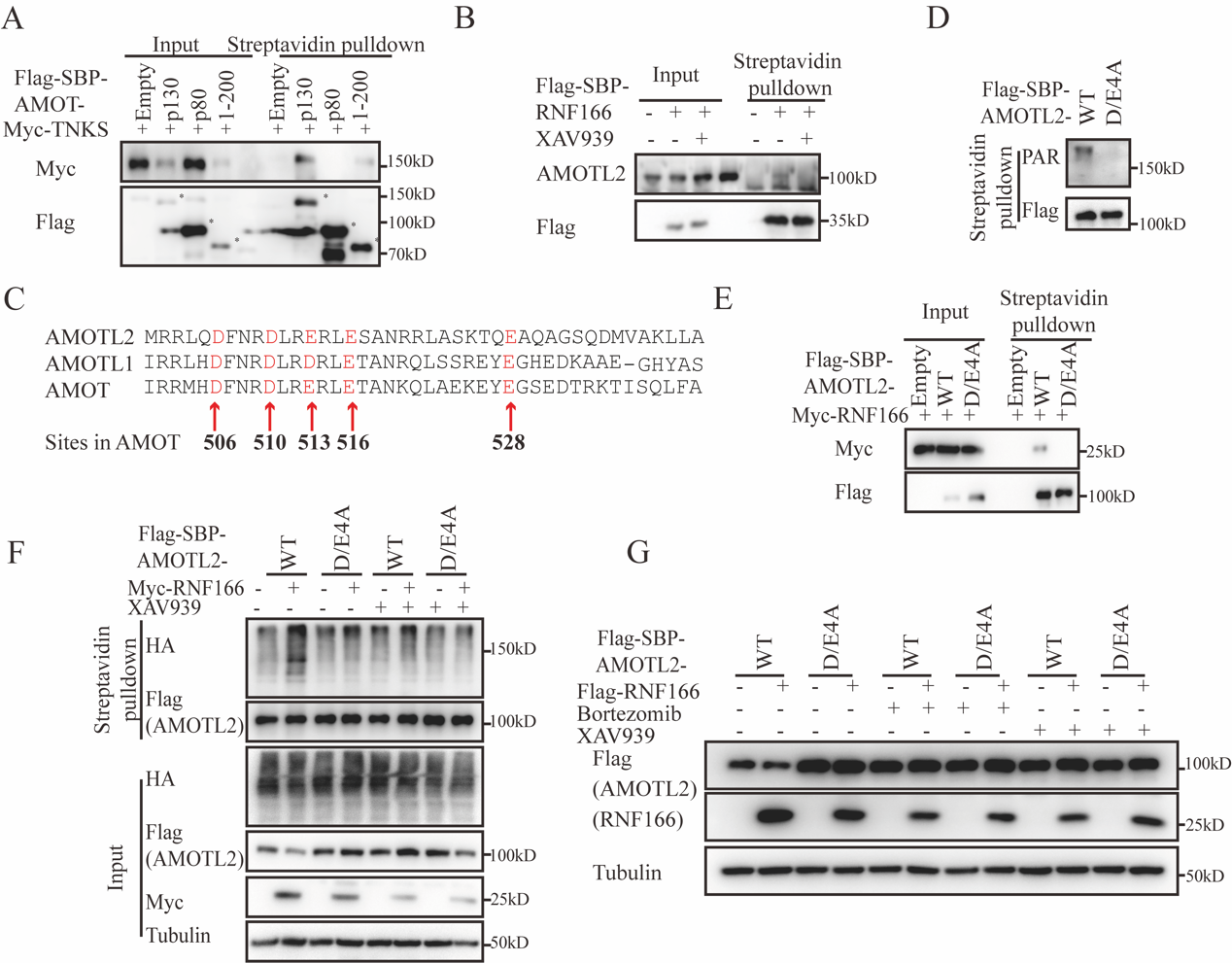
**

**Fig. S6: RNF166 recognizes PARsylated Motins.**

(A) AMOT^1-200^, instead of AMOTp80, effectively binds to tankyrase. Variants of AMOT are marked with asterisks. (B) Effect of XAV939 on disrupting RNF166 binding to AMOTL2. (C) Comparison of conserved amino acids in the Motins (region of 501 - 542 in AMOTp130) by ClustalW. Possible PARsylated sites are marked in red font. The numbers below correspond to the amino acid sites in AMOTp130. AMOTL2 with four alanine substitutions (D/E4A) results in loss of PARsylation (D), prevention of binding to RNF166 (E), resistance to protein ubiquitination by RNF166 (F), and insensitivity to bortezomib and XAV939 (F, G).


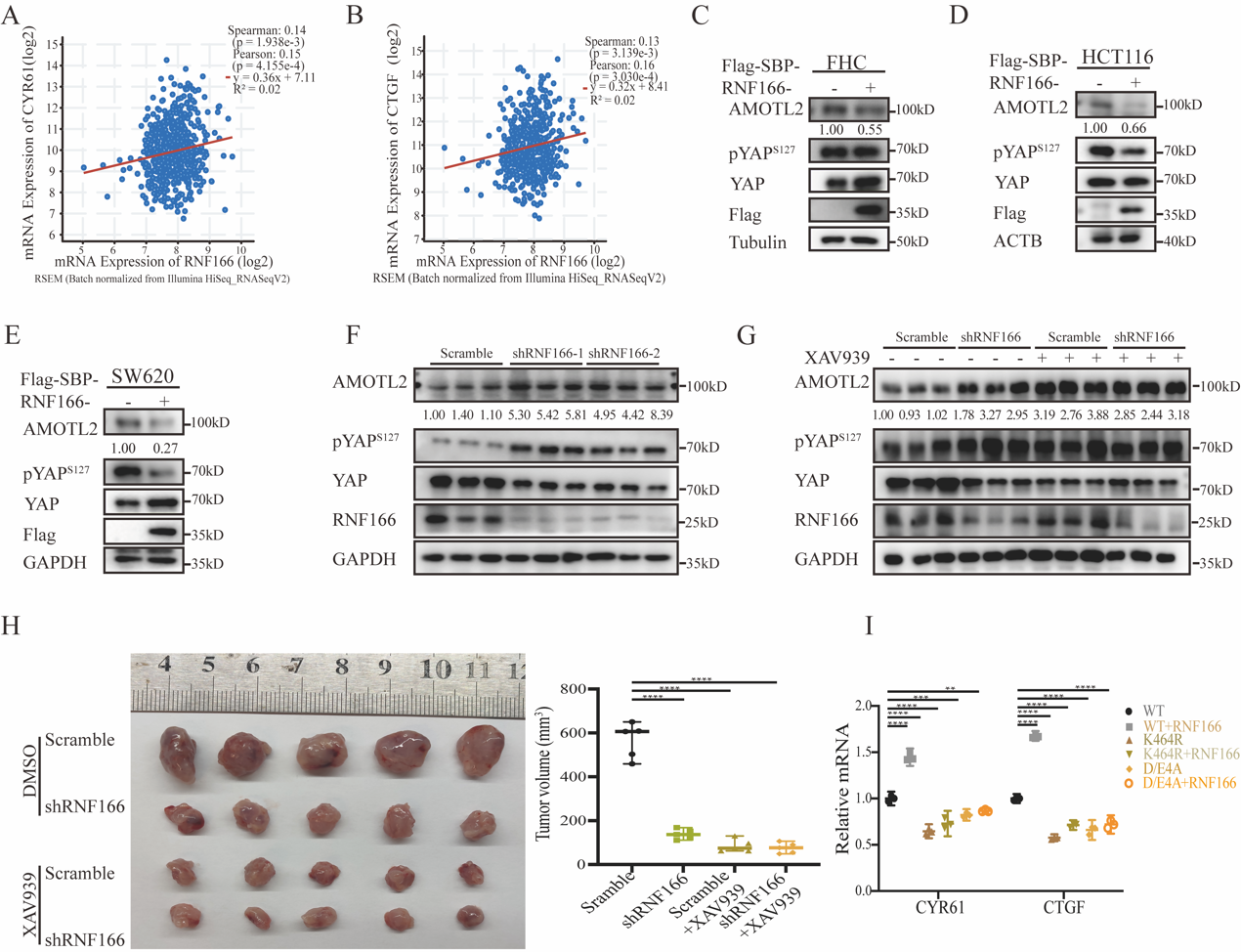


**Fig. S7: The Hippo pathway is regulated by RNF166.**

The expression of RNF166 is positively correlated with the expression of CYR61 (A) and CTGF (B), as shown by cBioPortal. (C, D, and E) Stably overexpressing RNF166 significantly affects Motins stability and activates YAP in FHC (C), HCT116 (D), and SW620 (E) cells. (F) RNF166 knockdown affects AMOTL2 protein levels and Hippo pathway in xenograft tumors. XAV939 treatment affects regulation of AMOTL2 protein levels and Hippo pathway (G) and total tumor volume (H) by RNF166 in HCT116 cell-derived xenografts. These tumors were resected and measured at the final timepoint, with 5 mice per cohort. (I) Mutations at the AMOT ubiquitination or PARsylation sites in 293T cells prevent the expression of target genes induced by RNF166 overexpression.

**Supplementary table**

**Supplementary table 1: The interacting proteins of RNF166 from BioPlex**

| **A** | **B** | **Networks** |
| --- | --- | --- |
| TNKS | RNF166 | BioPlex_3_0, BioPlexHCT_1_0 |
| RNF166 | TNFAIP6 | BioPlex_3_0 |
| RNF166 | XAF1 | BioPlex_3_0 |
| UBB | RNF166 | BioPlex_3_0 |
| TNKS2 | RNF166 | BioPlex_3_0 |
| PARP14 | RNF166 | BioPlexHCT_1_0 |
| PARP9 | RNF166 | BioPlexHCT_1_0 |
| RNF166 | ACP2 | BioPlex_3_0 |
| RNF166 | PLPP4 | BioPlex_3_0 |
| RNF166 | PARP11 | BioPlex_3_0 |
| RNF166 | BOLL | BioPlex_3_0 |
| RNF166 | CCDC85A | BioPlex_3_0 |
| NF2 | RNF166 | BioPlex_3_0 |
| KIF1A | RNF166 | BioPlex_3_0 |
| RNF166 | TMEM192 | BioPlex_3_0 |
| MPDZ | RNF166 | BioPlex_3_0 |
| RNF166 | CGA | BioPlex_3_0 |
| RNF166 | PARP12 | BioPlex_3_0 |
| RNF166 | TNFAIP3 | BioPlex_3_0 |
| RNF166 | AQP1 | BioPlex_3_0 |
| RNF166 | TIPARP | BioPlex_3_0 |
| RNF166 | CLEC4E | BioPlex_3_0 |
| RNF166 | LTBR | BioPlex_3_0 |
| PATJ | RNF166 | BioPlex_3_0 |
| RNF166 | TNFRSF13B | BioPlex_3_0 |
| DDI2 | RNF166 | BioPlexHCT_1_0 |
| RNF166 | C11orf24 | BioPlex_3_0 |
| BRCC3 | RNF166 | BioPlexHCT_1_0 |
| RNF166 | CACNG5 | BioPlex_3_0 |
| RNF166 | CHD1L | BioPlex_3_0 |
| RNF166 | ARMC10 | BioPlex_3_0 |
| RNF166 | DEFB136 | BioPlex_3_0 |
| AMOTL1 | RNF166 | BioPlex_3_0 |
| BMP2K | RNF166 | BioPlex_3_0 |
| RNF166 | PLPPR1 | BioPlex_3_0 |
| BABAM2 | RNF166 | BioPlexHCT_1_0 |
| RNF166 | EVA1B | BioPlex_3_0 |
| TJP1 | RNF166 | BioPlex_3_0 |
| RNF166 | APOC2 | BioPlex_3_0 |
| SNX3 | RNF166 | BioPlexHCT_1_0 |
| HLTF | RNF166 | BioPlex_3_0 |
| NUP58 | RNF166 | BioPlex_3_0 |
| RNF114 | RNF166 | BioPlex_3_0, BioPlexHCT_1_0 |
| SCRN1 | RNF166 | BioPlexHCT_1_0 |
| AMOT | RNF166 | BioPlex_3_0 |
| NUP35 | RNF166 | BioPlex_3_0 |
| NUP54 | RNF166 | BioPlex_3_0 |
| PARP1 | RNF166 | BioPlex_3_0 |
