# Supplementary material for: RNF166 promotes colorectal cancer progression by recognizing and destabilizing poly-ADP-ribosylated angiomotins
Source: Cell Death Dis. 2024 Mar 13;15(3):211. doi: 10.1038/s41419-024-06595-9 (PMC10937711; doi:10.1038/s41419-024-06595-9)
Supplement: Supplementary file 3 — Original Data File [file 41419_2024_6595_MOESM3_ESM.docx]

**Original western blots**

Figure 2B

Figure 2C

Figure 2D

Figure 2E

Figure 3A

Figure 3B

Figure 3C

Figure 3D

Figure 3E

Figure 3F

Figure 3G

Figure 3H

Figure 3I

Figure 3K

Figure 3L

Figure 3M

Figure 3N

Figure 4B

Figure 4C

Figure 4D

Figure 4E

Figure 4F

Figure 4G

Figure 4H

Figure 4I

Figure 4J

Figure 4K

Figure 4L

Figure 4M

Figure 4N

Figure 4O

Figure 5B

Figure 5C

Figure 5D

Figure 5E

Figure 5F

Figure 6E

Figure 6F

Figure 7E

Figure 7F

Supplementary Figures

Figure S1A

Figure S1B

Figure S1C

Figure S1D

Figure S1E

Figure S1F

Figure S3A

Figure S5D

Figure S5E

Figure S6A

Figure S6B

Figure S6D

Figure S6E

Figure S6F

Figure S6G

Figure S7C

Figure S7D

Figure S7E

Figure S7F

Figure S7G
